# Physiological Adaptations to Training in Competitive Swimming: A Systematic Review

**DOI:** 10.1515/hukin-2015-0120

**Published:** 2015-12-30

**Authors:** Mário J. Costa, Govindasamy Balasekaran, J. Paulo Vilas-Boas, Tiago M. Barbosa

**Affiliations:** 1 Sports Science Department, Polytechnic Institute of Guarda, Portugal; 2 National Institute of Education, Nanyang Technological University, Singapore; 3 Faculty of Sport Sciences, LABIOMEP, University of Porto, Portugal; 4 Research Centre in Sports, Health and Human Development, CIDESD, Portugal; 5 Centre of Research, Education, Innovation and Intervention in Sport, Portugal

**Keywords:** physiology, longitudinal, studies, exercise, swimming

## Abstract

The purpose of this systematic review was to summarize longitudinal studies on swimming physiology and get implications for daily practice. A computerized search of databases according to the PRISMA statement was employed. Studies were screened for eligibility on inclusion criteria: (i) present two testing points; (ii) on swimming physiology; (iii) using adult elite swimmers; (iv) no case-studies or with small sample sizes. Two independent reviewers used a checklist to assess the methodological quality of the studies. Thirty-four studies selected for analysis were gathered into five main categories: blood composition (n=7), endocrine secretion (n=11), muscle biochemistry (n=7), cardiovascular response (n=8) and the energetic profile (n=14). The mean quality index was 10.58 ± 2.19 points demonstrating an almost perfect agreement between reviewers (K = 0.93). It can be concluded that the mixed findings in the literature are due to the diversity of the experimental designs. Micro variables obtained at the cellular or molecular level are sensitive measures and demonstrate overtraining signs and health symptoms. The improvement of macro variables (i.e. main physiological systems) is limited and may depend on the athletes’ training background and experience.

## Introduction

Competitive swimming is one of the popular sports to be researched extensively. The aquatic environment presents specific challenges for humans. Hence, researchers are constantly willing to have a deeper insight into human performance in water. Cross-sectional and longitudinal designs have been selected to link swimming performance with physiological and biomechanical determinant factors. Cross-sectional studies involve the observation of a population, or a representative subset, at one single point in time. Such studies may be used to describe some distinctive features of the population or may support some sort of causality between variables. Longitudinal designs carry out a series of observations more than once on members of the study population over a period of time. The repeated and subsequent measurements throughout time can be represented by a dataset based on a three-dimensional axis (Van der Kamp and Bijleveld, 1998):

(1) Yijt=(i=1,2,,N;j=1,2,..,M;t=1,2,..,T),

where ijt represents the axis, N represents the number of subjects, M represents the variables and T represents the time measurements. Thus, subjects representative of an entire longitudinal sample are evaluated with the same experimental procedures, in a certain number of occasions throughout a timeframe. With this approach, it is possible to analyze the effect between the variables and how these variables, isolated (i.e., bivariate analysis) or in combination (i.e., multivariate analysis), contribute to sports performance.

The training programs, which are well planned with the appropriate physiological stimulus, are an effective way to disturb homeostasis and adapt the organism to subsequent improvements. Based on this reasoning, a large amount of evidence has been produced in the last few decades, enlightening us about the relationships between sports performance and training. One of the first reviews about training interventions in swimming was conducted by Lavoie and Montpetit (1986). The authors described the effects of training on energetic, respiration and cardiovascular variables as well as body composition. A few findings on health-related fields, such as muscle biochemistry, endocrinology and temperature regulation were also mentioned. Besides that, in the 1970s, 1980s and 1990s, most of the physiological research focused on “macro”-variables (i.e., main physiological systems, such as cardiorespiratory responses); research on “micro”-variables (i.e., at a cellular and molecular level) is now the main focus of several scientific teams, and it is a trend to be maintained in the next years as it happens in other competitive sports. However, to the best of our knowledge, no update about the recent advancements regarding the physiological adaptations (i.e., chronic adaptations assessed with longitudinal designs) to training has been done since then. The review papers published over the last 20 years had a strong focus on physiology and/or biomechanical acute responses in cross-sectional studies (Barbosa et al., 2010; Smith et al., 2002; Toussaint and Beck, 1992). On top of that, systematic reviews published in the meantime were about one or a couple of very specific training topics (Aspenes and Karlsen, 2012; Costa et al., 2012). Since most of the field practitioners are not used to work or consider these minor physiological aspects, there is a need to congregate pieces of evidence and clarify the relationship between dose response and training.

The purpose of this systematic review was to summarize the state of the art about the blood, hormonal, enzymatic, cardiovascular and energetic adaptations to swim training. The existence of mixed findings between studies dealing with physiological data within the season was hypothesized. This paper was largely restricted to research involving mature college-aged elite swimmers of both genders, and this should be kept in mind when considering the research-based points made.

## Material and Methods

Methodological procedures considering the standards for systematic reviews according to the PRISMA statement were employed (Moher et al., 2009). Expert researchers conducted the process to fulfil the suggestions and guidelines, such as (McGowan and Sampson, 2005) (i) the need of transparency (readers should be able to verify that the review is not open to bias) and (ii) reproducibility (readers and other researchers should be able to replicate the methods and arrive at the same results).

A search was conducted of literature dating from January 1st of 1970 until December 31th of 2013 using electronic literature databases (PubMed, ISI Web of Knowledge, Index Medicus, MEDLINE, Scopus, SPORTDiscus). Studies were identified using the following key terms individually and/or combined: “swimming”, “swimming effects”, “training effects”, “seasonal variations”, physiology and swim training”, “blood and swim training”, “cardiovascular and swim training”, “hormonal and swim training”, “enzymatic and swim training”, “energetics” and “swim training”. Two independent searches produced two different lists of publications that were then consolidated into one single list. The results were initially screened according to the title to exclude any obviously irrelevant articles. Potential hits that met the inclusion criteria were searched thereafter. When necessary, attempts were made to contact the authors to obtain the missing paper.

### Inclusion and Exclusion Procedures

The reduced evidence on other cohorts (youth or masters swimmers) when comparing them with the elite cohort led us to restrict the review. Included studies were focused on longitudinal interventions on physiology of mature college-aged elite swimmers. The excluded studies were (i) studies not having at least two testing points with the same subjects, (ii) studies based on other swimming topics rather than physiology, (iii) studies using other chronological ages (e.g., children, age-group or masters) instead of adult elite swimmers or other competitive levels, and (iv) case studies or those with a small sample size, which may compromise the sample power and external validity of the findings (e.g., *N* < 5).

In respect to the research question, relevant studies were categorized in five main groups: (i) blood composition, (ii) endocrine secretion, (iii) muscle biochemistry, (iv) cardiovascular response, and (v) energetic profile. The information extracted from the included studies was based on (i) design and setting, (ii) sample characteristics, (iii) aim of the intervention, and (iv) major findings.

### Quality Assessment

All relevant studies underwent a formal methodological assessment by two independent reviewers. Since there is no validated quality assessment tool suitable for this kind of field interventions (i.e., sports performance), the quality index score of each paper was calculated with a checklist described earlier (Downs and Black, 1998). This checklist presents a large range of scoring profiles: reporting, external validity, bias, confounding and power. In each profile, all items received rating scores, where the maximum possible score to obtain from the index was 32 points. When necessary or appropriate, disagreements between reviewers were solved by discussion and consensus. The degree of agreement in scoring procedure was obtained based on the Kappa index (*K*) and thresholds interpreted according to Landis and Koch’s (1977) suggestion, where there is (i) no agreement if K ≤ 0, (ii) poor agreement if 0 < K ≤ 0.19, (iii) fair agreement if 0.20 < K ≤ 0.39, (iv) moderate agreement if 0.40 < K ≤ 0.59, (v) substantial agreement if 0.60 < K ≤ 0.79, and (vi) almost perfect agreement if 0.80 < K ≤ 1.00.

## Results

**Figure 1:**
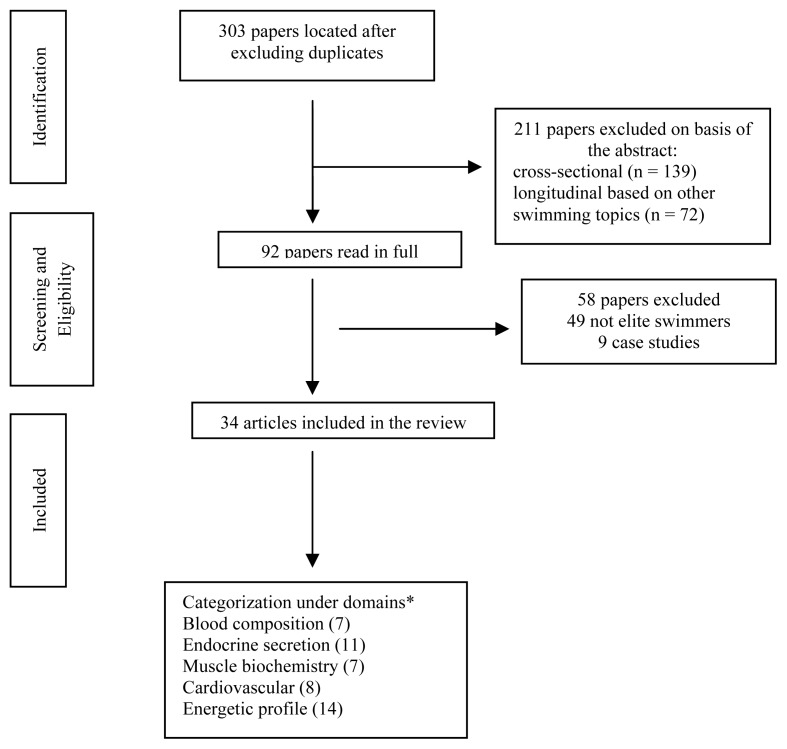
Search strategy; * eight studies categorized in several domains

Our search identified 303 potential relevant papers of which 269 did not meet the inclusion criteria. The reasons for exclusion were being cross-sectional (139 studies), longitudinal focused on other topics (72 studies), participants from other chronological ages or competitive level (49 studies) and case studies (9 studies) (Figure 1). A total of 34 studies were considered for further analysis. From these, the earliest one was published in April of 1980 (Rushall and Busch, 1980) and the most recent in December of 2013 (Costa et al., 2013a).

Studies were examined for each category according to their reported data: (i) blood composition (7 studies), (ii) endocrine secretion (11 studies), (iii) muscle biochemistry (7 studies), (iv) cardiovascular response (8 studies), and (v) energetic profile (14 studies). Since 8 studies reported various data, they were included in several physiological topics. Hence, the total number of papers in categorization does not match the sum of the partial number by categories. The topic with a higher number of papers and probably with a more consistent body of knowledge is the energetic profile. Muscle biochemistry is the topic with reduced evidence or at least with fewer papers published.

The quality index had a mean score of 11.04 ± 2.04 points (range: 8 to 19). The reliability between both reviewers showed an almost perfect agreement (*K* = 0.93) in the scoring procedure. The studies scored similarly in their reporting style. All studies performed better in reporting items than in external validity, bias, confounding, and power. Items related with the aim and hypothesis description, sample characteristics, estimates of random variability, and the definition of the outcomes to be measured were the ones more clearly defined in the majority of the included studies. The higher ratings of quality were found in the blood composition category (12.14 ± 3.45 points). Only one study calculated the sample power to detect a clinically meaningful effect (Rama et al., 2013). At least in two studies, the losses of subjects to follow up were reported (Costa et al., 2013a; Costa et al., 2013b).

Seven studies monitored changes in the blood composition of elite swimmers during training. The overall quality scores ranged between 8.5 to 19 points (average score for all papers: 12.14 ± 3.45 points; average score for papers published in the last 10 years: 14.33 ± 4.16 points). Interventions were mostly focused on hematological or immune system components.

Eleven studies analyzed hormonal variations during heavy and intense training phases of the season. The overall quality scores ranged between 8 to 11.5 points (average score for all papers: 9.70 ± 1.03 points; only one paper found in the last 10 years with a quality score of 10 points). The hormonal markers often collected through blood samples were cortisol, testosterone and the catecholamines.

Seven studies monitored changes in muscle biochemistry of elite swimmers during different training periods. The overall quality scores ranged between 9 to 11 points (average score for all papers: 10.29 ± 0.81 points; no paper was found in the last ten years). The experimental approaches used muscle biopsy or blood sample assessment to collect enzymatic data.

Eight studies analyzed changes in the cardiovascular responses of elite swimmers. The overall quality scores ranged between 8.5 to 12 points (average score for all papers: 10.06 ± 1.24 points; average score for papers published in the last 10 years: 11.20 ± 1.04 points). The studies dealt with heart rate or blood pressure variability through several training periods.

Fourteen studies monitored changes in the energetic profile of elite swimmers. The overall quality scores ranged between 8.5 and 14 points (average score for all papers: 11.32 ± 1.58 points; average score for papers published in the last 10 years: 12.60 ± 1.28 points). Interventions were generally aimed at assessing the lactate threshold (LT), peak lactate (La_peak_) and maximal oxygen uptake (VO_2max_).

## Discussion

The focus of this investigation was to summarize evidence about the blood, hormonal, enzymatic, cardiovascular and energetic adaptations to swim training. Controversial findings were found in the literature mostly due to the diversity of the experimental designs. Reports on micro variables showed links between overreaching/overtraining and the poor health status of the swimmers. Papers dealing with macro variables justified the physiological changes based on training background and experience.

### Blood profile

The swimmers’ blood composition monitoring (i.e., hematological or immune parameters) may indicate poor dietary intake or an irrational training load, leading to increased susceptibility to illness (Morgado et al., 2012).

Four studies examined the variation of hematological parameters during long-lasting periods. Rushall and Busch (1980) showed that haemoglobin values of elite swimmers were reduced during hard training phases of the season and increased during the reduced workload of tapering. In a 14-week training intervention, Santhiago et al. (2009) verified that hematocrit and mean corpuscular volume diminished during the endurance phase (men: 5.8 and 7.2%; women: 11.6 and 6.8%, respectively) and increased in the intensity increased phase (men: 7.2 and 6.0%; women: 7.4 and 5.2%, respectively) of the season. Mujika et al. (1998) demonstrated increases in hemoglobin and mean corpuscular volume of highly trained swimmers after 12 weeks of intense training followed by 4 weeks of taper. Conversely, serum ferritin, hemoglobin, erythrocyte number, hematocrit, and mean red cell volume showed no changes after 4 weeks of intensified training (Mackinnon et al., 1997). The total duration of the interventions, the number of testing points, and the possible difference between training loads constitute some of the reasons for this discrepancy between results. The applicability of the findings highlights the importance of regular blood sample analysis in each stage of the season mostly to adjust training loads accordingly.

Understanding the effects of training on the immune system should be considered an additional measure to help coaches detect the poor health status of their swimmers. Three studies reported findings on immune parameters or respiratory infections during long periods of swim training. Morgado et al. (2012) observed changes in the number of monocytes (from 468 to 429 cells/μl), neutrophils (from 4536 to 3929 cells/μl), and dendritic cells (from 49 to 56 cells/μl) after 24 weeks of training with high volume and intensity. Glesson et al. (2004) found no significant differences in T-lymphocyte function after extended periods of training at the elite level. Rama et al. (2013) determined that upper respiratory episodes (67%) clustered with the period of high intensity and volume training were accompanied by a decrease in the percentage of natural killer cells in the immune system (ranging from 17 to 27% of loss according with the specific stage of the season). Currently, the scarce number of studies on this topic leaves unclear indications of a real trend and therefore makes it more challenging to provide reliable evidence to practitioners. Probably, regularly using blood samples from top-level swimmers should be a must in monitoring the response to training loads. Beyond investigating the effects of different training loads on the immune response, we should try to understand how supplementation may improve the immune status according to the training load.

### Endocrine Secretion

Vigorous exercise has a short- and a long-term effect on the endocrine responses of individuals (Viru et al., 1992). Among the hormonal markers often collected through blood samples there are cortisol, testosterone and catecholamines (including epinephrine and norepinephrine). Eleven studies were published about the effects of training on the endocrine response of elite swimmers. From those, nine studies analysed hormonal changes in different time points of the season, and two studies correlated hormonal training effects with performance.

Kirwan et al. (1988) observed that serum cortisol significantly increased (from ~17.5 to ~20.6 μg/dl) by doubling the swimmers’ training distance (~4 to ~9 km/day) while maintaining the intensity at approximately 95% of maximal oxygen uptake. Costill et al. (1991) determined an increase in cortisol (from 19 to 24 μg/dl) and a decrease in testosterone (from ~8 to 6 ng/ml) during the period of increased training volume. Tyndall et al. (1996) observed higher resting plasma cortisol concentrations in women compared to men at the beginning of the season when training volume averaged 5.5 km/day; albeit, Hooper et al. (1993) found unchanged norepinephrine or cortisol concentrations at five testing points during a 6-month season. Similar statistically non-significant changes were observed by Hakinnen et al. (1989) in the concentrations of serum testosterone (from 30.9 to ~29 nmol/l) and cortisol (from 0.70 to ~66 μmol/l) at the first and most intensive training period of the year. Although hormonal values are in similar range when comparing various samples of swimmers, remaining studies fail to detect significant hormonal changes in response to training (Flynn et al., 1994; Mackinon et al., 1997; Mujika et al., 1996a; Mujika et al., 1996b). Despite being consensual that any abrupt changes in the training load will trigger an endocrine response, the small sample sizes and the discrepancy between testing points can help explain the trivial hormonal changes in some reports.

With regard to the impact on race times, Hooper et al. (1999) observed that the change in plasma norepinephrine concentration predicted the change in swim time with tapering (*r*^2^ = 0.82) by itself. Similarly, Atlaoui et al. (2006) determined that the percentage changes in performance during reduced training phases had significant relationships (*r* = 0.60) with norepinephrine levels. Athletes with overtraining symptoms after several weeks of high-intense training were found to have decreased levels of norepinephrine (Mackinnon et al., 1997). Despite few evidence-based practices, high norepinephrine concentrations seem to be important to avoid symptoms of overtraining and maintain the athletes’ health for competition.

In terms of daily practice, the regular assessment of hormonal concentrations during heavy and intense training phases should be a criterion. Future researchers need to spend more effort in understanding hormonal behaviour with respect to overtraining and burnout, critically analyzing the number of training sessions per week, the duration of those sessions, and the external load (i.e., high volume and intensity).

### Muscle biochemistry

Muscle metabolism has been another point of interest in swimming physiology. Enzymes are known as body catalysts that have a particular action in neuromuscular response. Seven studies have focused on how enzymatic activity responds to prolonged swim training.

Houston et al. (1981) found significant increases of hexokinase (from ~1.0 to ~1.5 μmol/min/g), phosphorylase (from ~8.2 to ~10.0 μmol/min/g), phosphofructokinase (from ~26.1 to ~35.0 μmol/min/g) and succinate dehydrogenase (from ~5.6 to ~7.1 μmol/min/g) activity from the deltoid muscle when increasing training volume or intensity. A similar finding was reported by Costill et al. (1985) showing an increased activity in citrate synthase (from ~34 to ~40 μmol/min/g) in the deltoid muscle after increased training volume. Two other studies determined increases in creatine kinase (CK) levels after heavy training periods (Burke et al., 1982; Kirwan et al., 1988). These chemical adaptations after intense training sets may prevent the muscle from contracting and thus help the body carry out the desired action. However, recovery periods help restore muscle chemical function and reduce enzymatic levels suggesting that peripheral adaptation may occur and improve muscle ability for further efforts (Lavoie and Montpetit, 1986). In fact, two other studies showed unchanged CK levels after seasonal periods with increased training volume followed by taper phases (Millard et al., 1985; Mujika et al., 1996b). Although there were no statistically significant differences, the results of Mujika et al. (1996b) showed slight increases in CK concentrations after intense training which corresponded to declines in competition performance before taper. Inconsistent results may also rely on the different methods to assess muscle biochemistry (muscle biopsy vs. blood samples) and on subsequent analysis. In most of the interventions, the CK measurement was made on the basis of blood serum as markers of muscle damage. As CK peaks several hours after the cessation of training, as far as we know, the reported time point of drawing the blood in such interventions was 45 h after intense exercise cessation (Millard et al., 1985). Remaining interventions (Costill et al., 1991; Mujika et al., 1996b) made blood sampling in the morning after a light training session on the previous day and an intensive training session two days before. The type of exercise performed may also explain mixed findings. It is well known that serum CK concentrations may change in response to eccentric exercise, particularly, intense eccentric exercise. Swimming is considered a non-eccentric exercise mode, due to the reduced body weight effect associated to buoyancy and to the lack of ballistic actions. Nevertheless, there is evidence of relevant co-contraction muscular intervention modes, possibly implying not only joint stabilization, but also eccentric actions of the antagonist muscles.

Overall, evidence supports that regular measurement of enzymatic levels during a season, along with other physiological variables, may help determine overtraining symptoms in swimmers. Further research should try to provide insight in the chronic effects of dry-land strength training and how this could have a positive effect on muscle biochemistry during hard training phases and recovery periods.

### Cardiovascular response

The cardiovascular assessment has been, for a long time, an important component for training monitoring. Coaches still select on a daily basis cardiovascular measures to monitor and prescribe training intensity.

Eight papers studied how cardiovascular measures were affected by swim training. Houston et al. (1981) observed decreases in the peak heart rate recorded during treadmill running and tethered swimming (from ~204 to ~193 bpm and from ~172 to ~165 bpm, respectively) after swim training. Sharp et al. (1984) showed unchanged heart rate values during several testing points through the season, but the swimmers were able to swim faster covering a 200-m distance freestyle. Despite these reports, recent interventions showed unchanged values in the peak heart rate (Anderson et al., 2006; Psycharakis, 2011) and the resting heart rate (Atlaoui et al., 2007; Costill et al., 1991; Flynn et al., 1994; Kirwan et al., 1988) over a swimming season. The significant changes reported by Houston et al. (1981) may be explained by the training background of the subjects (only four of the ten swimmers recruited had trained during the preceding four months). It is known that untrained subjects taking part in any vigorous training program have a higher odd to show significant changes in the cardiovascular response than trained counterparts (Earle and Baechle, 2004). Hence, a closer inspection is needed to clarify the usefulness of heart rate measures to monitor the effects of external training loads.

The two remaining studies on this topic failed to find the effects of swim training on blood pressure. Kirwan et al. (1988) analyzed the cardiovascular responses of 12 male collegiate swimmers during 10 days of intense training and determined that resting systolic blood pressure was not affected when training load increased from ~4,200 to ~8.900 m/day. Flynn et al. (1994) found no differences in the mean blood pressure of swimmers through a full collegiate season. At some point of their competitive career, the absence and/or tenuous changes observed in adult swimmers may result from the inability to induce substantial adaptations in the stroke volume, mostly due to heart size, that has already reached is maximum size and shape (Kubukeli et al., 2002). Hence, there is a trend to see unchanged blood pressure levels after any type of swim training through the season.

Despite cardiovascular monitoring being a straightforward procedure, evidence suggests that it is not sensitive enough. For instance, heart rate response to exercise can be quite variable depending on several external factors and not only on exercise intensity. Hence, some care should be taken if the heart rate or the blood pressure is selected to control training effects.

### Energetic profile

Several changes that might occur on the energetic profile are determined by the nature of the training stimulus. Training volume, intensity and frequency are the components used by coaches to work the peak form status of their athletes for the most important periods of the season (Mujika, 1998). According to this, several research groups have assessed the effect of a training load on energetics. Fourteen studies were included on this chapter showing evidence on LT velocity, La_peak_ and/or VO_2max_.

Six studies found a small change in the LT velocity within or between swimming seasons (Anderson et al., 2006; Costa et al., 2013b; Pyne et al., 2001; Ryan et al., 1990; Santhiago et al., 2009; Sharp et al., 1984). Three of those studies showed that an increase in training volume during the middle of the season did not significantly change the LT velocity in the following testing occasions (Costa et al., 2013b; Ryan et al., 1990; Sharp et al., 1984). Findings are consistent and show that an unchanged LT from a given point of the season seems to be an individual limitation and not a training issue. In fact, Faude et al. (2008) determined that high training volume had no advantage compared with high-intensity training of lower volume in improving the LT of elite swimmers. There are reports that the competitive level may also determine the range of change in the LT velocity during one competitive season (Costa et al., 2012). The effects of high training volume mostly used at the start of the season promote decreases in glycogen stores. This phenomenon results in lower lactate concentrations at similar effort intensities through the training season (Costill et al., 1991).

Studies on La_peak_ showed that training characteristics may determine the trend in La_peak_ variation. Costill et al. (1991) observed a reduction in La_peak_ values after 365.8 m submaximal swim (from ~13 to ~8 mmol/L) in response to increased training volume during the first 8 weeks of the season. Wakayoshi et al. (1993) reported that six months of aerobic swim training was enough to significantly decrease La_peak_ after a 400 m swimming effort. In contrast, Termin and Pendergast (2000) determined increases (from 8.71 to 11.59 mmol/L) in the La_peak_ of swimmers included in a high-intensity training program over four consecutive seasons. Similarly, Costa et al. (2013a) observed increases in the La_peak_ after a maximal 200 m front crawl test (from ~10 to ~12 mmol/L) when training intensity increased from one season to another. While training with a more intense regimen, the muscle is forced into adaptations that allow the body to reach higher velocities at an increased oxygen debt and reduced muscle fatigue, improving anaerobic metabolism.

Few studies dealt with VO_2max_ assessment over a long-term training period. Houston et al. (1981) found significant increases in VO_2max_ during treadmill running (from ~ 3.7 to 4.1 l/min), but not during tethered swimming (unchanged at 3.3–3.4 l/min) after swim training. For Costa et al. (2013b), VO_2max_ of elite swimmers remained slightly unaltered (between 72 and 76 ml/kg/min) in the three testing points during one full season of training. Johns et al. (1992) also observed consistency in the VO_2max_ (between 3.5–3.2 l/min) of collegiate swimmers after a 10-day taper. On the contrary, Termin and Pendergast (2000) observed a 20, 9, 8 and 5% increase in VO_2max_ after each of the four consecutive swimming seasons of the program. The inconsistent findings can be explained by the training intensity and the total duration of the interventions. In the study of Houston et al. (1981), the 6.5 weeks of moderate and high intensity training were not sufficient to promote substantial adaptations in VO_2max_. Any attempt to induce meaningful adaptations may require a longer period of time. Simultaneously, the trivial changes of VO_2max_ reported by Costa et al. (2013b) are justified by the high competitive level of the swimmers recruited. Termin and Pendergast (2000) recruited US division I male swimmers. One might say that those were collegiate subjects with higher range of improvement in VO_2max_. Thus, VO_2max_ is highly dependent on the program characteristics and the subject’s training background. The capacity to transport and use oxygen can increase as a result of high intensity training, but this increase is dependent on the swimmer’s expertise and tends to decrease through consecutive seasons. This shows that the type and duration of training may elicit different adaptations regarding the transport and use of oxygen, as opposed to cardiac size and contraction already described in previous chapters.

Understanding the effects of training on the energetic pathways is more consistent than that of the remaining physiological variables. However, a deep understanding should be obtained of the effects of different training program characteristics (high versus low intensity and/or high versus low volume) on swimming strokes other than the front crawl.

### Quality assessment

As a tool to assess the quality of each paper, we used a checklist that provided a profile of the paper, alerting reviewers to its particular methodological strengths and weaknesses. The scoring checklist had already been identified as a reliable tool to assess the methodological quality not only of randomised controlled trials but also of non-randomised studies (Downs and Black, 1998). It has some items related to complex procedures such as randomization, blindness, the use of control group and/or practical effects. Since swimming teams have only a small number of caliber swimmers to be assessed, most of the times researchers recruit convenience samples, which prevents to extrapolate the result for all population. That is the reason why the interventions with more careful procedures and controlled environmental conditions (e.g. effects of swim training on blood composition) had better scoring rates. The remaining interventions fail to have a more “health” approach and then culminate with a lower scoring rate.

When scoring only the papers published in the last ten years, there is a trend toward an increase in the scoring rate for all categories. The reviewers’ knowledge about methodological quality, the journals’ policy about paper quality, and the more advanced experimental procedures can be found as reasons for this increase in scores in the last couple of years. Hence, further studies in swimming should take those several methodological issues into consideration in the experimental setup in order to increase the quality of the interventions even more.

## Conclusions

The included studies about training interventions on swimming physiology had more expression in the energetic profile category (14 studies) followed by endocrine secretion (11 studies), cardiovascular response (8 studies), blood composition (7 studies) and muscle biochemistry (7 studies). The diversity of the experimental designs (e.g., follow-up periods, training components, gender and competitive levels) can explain inconsistent findings through a training season. The assessment of micro variables at the cellular or molecular level is quite a sensitive procedure, but extremely informative about overreaching/overtraining symptoms and the health status of the swimmers. Hence, the regular use of blood samples by medical prescription should be an additional measure applied by coaches in daily practice. Although the findings on macro-variables are more consistent, those physiological changes are dependent on the swimmers training background and experience. Some methodological inadequacies (i.e., absence of a control group or reduced sample sizes) can be observed in the literature. This should be overcome in future research to reduce the ambiguity of the results. The interpretation of marginal gains and their contribution to the final performance should not be neglected. Although some studies failed to find significant physiological changes, the tiny effect promoted by swim training may impair or improve competition performance. A deeper insight into the less studied areas is needed. Scientists have recently started to study the effects of swim training on health-related issues such as blood modifications and respiratory infections, but numerous doubts still remain.

## Figures and Tables

**Table 1:** Summary of longitudinal interventions on blood composition

Author	Quality Score	Design and setting	Sample characteristics	Intervention	Findings
Rushall and Busch (1980)	8.5	12 months	Elite swimmers	Effect of hard training following taper	Hb decreased with hard training and increased during taper.
Mackinnon et al. (1997)	9.5	4 weeks	Well-trained swimmers	Compare cohorts exposed to high training	Hb, hematrocrit, red blood cells remained unchanged.
Mujika et al. (1998)	11	16 weeks	Highly trained swimmers	Effect of hard training following taper	Hb increased.
Gleeson et al. (2004)	13	5 months	11 elite swimmers	Effects of hard training following taper	T-lymphocyte response remained unchanged.
Santhiago et al. (2009)	11	14 weeks	25 International level swimmers	Analyse annual changes	Hematocrit decreased during the endurance phase and increased after.
Morgado et al. (2012)	13	7 and 17 weeks	Competitive level swimmers	Effect of long term intensive training	Significant changes in neutrophils, monocytes and dendritic cells.
Rama et al. (2013)	19	12 months	Elite swimmers and controls	Track respiratory infections	Respiratory infections rise from decreases in nature killing cells.

Hb – hemoblobin

**Table 2:** Summary of longitudinal interventions on endocrine secretion

Author	Quality Score	Design and setting	Sample characteristics	Intervention	Findings
Hakkinen et al. (1987)	10.5	1 year	9 elite swimmers	Effects of prolonged endurance training	Cor and Test showed non-significant changes.
Kirwan et al. (1988)	9	2 weeks	12 college swimmers	Effects of high volume and high intensity	Increases in Cor Epi and Nor.
Costill et al. (1991)	10.5	25 weeks	24 college swimmers	Effects of high volume	Increases in Cor and decreases in Test.
Hooper et al. (1993)	8	6 months	14 elite swimmers	Detecting overtraining markers	Unchanged Cor and Nor and decreases in Epi.
Flynn et al. (1994)	9	18 weeks	5 college swimmers	Detecting overtraining markers	Unchanged Cor and decreases in Test.
Mujika et al. (1996)	11	16 weeks	8 elite swimmers	Intense training followed by taper	Unchanged Cor, Test, Epi and Nor levels.
Mujika et al. (1996)	11.5	26 weeks	8 elite swimmers	Effects of annual training and taper	Cor remained unchanged.
Tyndall et al. (1996)	10	18 weeks	19 elite swimmers	Verify insulin action by hormonal changes	Cor or Test changes not affect insulin action after hard training.
Mackinnon et al. (1997)	9.5	4 weeks	24 elite swimmers	Effects of high volume	Nor was the best marker of overtraining symptoms.
Hooper et al. (1999)	10	2 weeks	10 elite swimmers	Effects of taper	High performance prediction based on Nor changes.
Atlaoui et al. (2006)	10	12 weeks	14 international and national swimmers	Associate endocrine levels with performance	Nor values were related with performance.

Cor – cortisol; Test – testosterone; Epi – epinephrine; Nor – norepinephrine.

**Table 3:** Summary of longitudinal interventions on enzymatic activity

Author	Quality Score	Design and setting	Sample characteristics	Intervention	Findings
Houston et al. (1981)	10	6.5 weeks	10 college swimmers	Effects of 2 types of training	Increases in HEX, PHOSK, PHOSL e SD activity.
Burke et al. (1982)	9.5	1 year	Elite swimmers	Hard training followed by taper.	Increases in CK until taper with following decreases.
Millard et al. (1985)	11	5 months	20 college swimmers	Hard training followed by taper.	Increases in CK until taper with following decreases.
Costill et al. (1985)	11	5 months	8 college swimmers	Hard training followed by detraining	Increases in PHOSK and PHOSL in deltoid but without significant losses during detraining.
Kirwan et al. (1988)	9	2 weeks	12 college swimmers	Increases in training intensity	Increases in CK levels.
Costill et al. (1991)	10.5	25 weeks	24 college swimmers	Increases in training volume	Increases in CS activity in deltoid and CK levels.
Mujika et al. (1996)	11	16 weeks	8 elite swimmers	Hard training followed by taper	CK levels remained unchanged.

CK - creatine kinase; HEX – hexokinase; PHOSK – phosphofructokinase; PHOSL – phosphorylase; SD - succinate dehydrogenase.

**Table 4:** Summary of longitudinal interventions on cardiovascular response

Author	Quality Score	Design and setting	Sample characteristics	Intervention	Findings
Houston et al. (1981)	10	6.5 weeks	10 college swimmers	Effects of two types of training	Decreases in HR_peak_.
Sharp et al. (1984)	8.5	6 months	12 college swimmers	Analyse changes in HR	Decreases in HR_peak_ only in the first two months of the season.
Kirwan et al. (1988)	9	2 weeks	12 college swimmers	Effects of taper	HR_rest_ remained unchanged. SBP remained unchanged.
Costill et al. (1991)	10.5	25 weeks	24 college swimmers	Effects of an increased volume	Decreases in HR_peak_ only in the first two months of the season.
Flynn et al. (1994)	9	18 weeks	5 college swimmers	Analyse effects of one season	MBP remained unchanged.
Anderson et al. (2006)	11.5	6 years	40 international and national level swimmers	Analyse within and between season changes	HR_peal_ remained unchanged.
Atlaoui et al. (2007)	10	7 weeks	13 international and national level swimmers	Relationship between HR and performance	HR_rest_ remained unchanged with no association with performance.
Psycharakis (2011)	12	6 months	17 international level swimmers	Analyse HR annual changes	HR_peak_ remained unchanged.

HR_peak_ – peak heart rate; HR_rest_ – resting heart rate; MBP- mean blood pressure; SBP – systolic blood pressure.

**Table 5:** Summary of longitudinal interventions on the energetic profile

Author	Quality Score	Design and setting	Sample characteristics	Intervention	Findings
Houston et al. (1981)	10	6.5 weeks	10 college swimmers	Effects of two types of training on VO_2max_	VO_2max_ remained unchanged in both programs.
Sharp et al. (1984)	8.5	6 months	12 college swimmers	Analyse changes in LT profile	Increases in LT at the first two months of the season.
Ryan et al. (1990)	10	6 months	14 college swimmers	Effects of high volume	Volume above 49.000 km/week did not change LT.
Costill et al. (1991)	10.5	25 weeks	24 college swimmers	Effects of high volume	Increases in LT and reductions in La_peak_.
Johns et al. (1992)	10	14 days	12 intercollegiate swimmers	Effects of taper	VO_2max_ remained unchanged.
Wakayoshi et al. (1993)	11	6 months	8 college swimmers	Aerobic training effects	Increases in LT and reductions in La_peak_.
Termin and Pendergast (2000)	11	4 years	22 US division I swimmers	Effects of an increased training program	Increases in La_peak_. And VO_2max._
Pyne et al. (2001)	12	8 months	12 world class swimmers	Analyse annual changes	Increases in LT.
Anderson et al. (2006)	11.5	6 years	40 international and national level swimmers	Analyse annual changes	Increases in LT within and between seasons.
Faude et al. (2008)	12	5 weeks	10 national level swimmers	Compare two types of training	Both high intensity and high volume increased LT.
Santhiago et al. (2009)	11	14 weeks	23 international level swimmers	Changes at different stages of the season	Only men swimmers improved LT.
Costa et al. (2012)	14	9 months	10 International and national level swimmers	Compare changes between cohorts	National swimmers improved in a higher range LT.
Costa et al. (2013^b^)	13	9 months	9 international and national level swimmers	Analyse annual changes	LT and VO_2max_ increased slightly.
Costa et al. (2013^a^)	14	2 years	12 international and national level swimmers	Analyse changes within and between seasons	LT showed non-significant increases within and between seasons. La_peak_ increased significantly.

LT – lactate threshold; La_peak_ – peak of blood lactate concentrations; VO_2max_ – maximal oxygen upt
